# The High Costs of Low-Grade Inflammation: Persistent Fatigue as a Consequence of Reduced Cellular-Energy Availability and Non-adaptive Energy Expenditure

**DOI:** 10.3389/fnbeh.2018.00078

**Published:** 2018-04-26

**Authors:** Tamara E. Lacourt, Elisabeth G. Vichaya, Gabriel S. Chiu, Robert Dantzer, Cobi J. Heijnen

**Affiliations:** Neuroimmunology Laboratory, Symptom Research Department, The University of Texas MD Anderson Cancer Institute, Houston, TX, United States

**Keywords:** cytokines, metabolism, effort, motivation, energy balance, chronic fatigue syndrome, cancer-related fatigue

## Abstract

Chronic or persistent fatigue is a common, debilitating symptom of several diseases. Persistent fatigue has been associated with low-grade inflammation in several models of fatigue, including cancer-related fatigue and chronic fatigue syndrome. However, it is unclear how low-grade inflammation leads to the experience of fatigue. We here propose a model of an imbalance in energy availability and energy expenditure as a consequence of low-grade inflammation. In this narrative review, we discuss how chronic low-grade inflammation can lead to reduced cellular-energy availability. Low-grade inflammation induces a metabolic switch from energy-efficient oxidative phosphorylation to fast-acting, but less efficient, aerobic glycolytic energy production; increases reactive oxygen species; and reduces insulin sensitivity. These effects result in reduced glucose availability and, thereby, reduced cellular energy. In addition, emerging evidence suggests that chronic low-grade inflammation is associated with increased willingness to exert effort under specific circumstances. Circadian-rhythm changes and sleep disturbances might mediate the effects of inflammation on cellular-energy availability and non-adaptive energy expenditure. In the second part of the review, we present evidence for these metabolic pathways in models of persistent fatigue, focusing on chronic fatigue syndrome and cancer-related fatigue. Most evidence for reduced cellular-energy availability in relation to fatigue comes from studies on chronic fatigue syndrome. While the mechanistic evidence from the cancer-related fatigue literature is still limited, the sparse results point to reduced cellular-energy availability as well. There is also mounting evidence that behavioral-energy expenditure exceeds the reduced cellular-energy availability in patients with persistent fatigue. This suggests that an inability to adjust energy expenditure to available resources might be one mechanism underlying persistent fatigue.

## Introduction

Chronic or persistent fatigue is a common, debilitating symptom of several diseases. It is one of the most frequently reported symptoms of cancer and cancer treatment (Servaes et al., 2002; Abrahams et al., 2016) and is highly prevalent in several chronic diseases, such as multiple sclerosis, diabetes, and rheumatoid arthritis (Wolfe et al., 1996; Drivsholm et al., 2005; Induruwa et al., 2012; Sanoobar et al., 2015). In addition, it is the hallmark symptom of chronic fatigue syndrome, a condition in which severe persistent fatigue is experienced in absence of a diagnosed disease (Fukuda et al., 1994; Afari and Buchwald, 2003). Persistent fatigue is distinct from acute fatigue. Acute fatigue is a healthy, adaptive response to physical or mental exertion, inducing metabolic signaling to prevent further energy consumption (Keyser, 2010). Acute fatigue typically resolves after rest or sleep. In contrast, persistent fatigue is often disproportional to exerted activities and is generally not completely alleviated by rest. No treatments for persistent fatigue have been approved by the US Food and Drug Administration, in part because the underlying mechanisms are still poorly understood.

Activation of inflammatory pathways has been suggested to underlie persistent fatigue in many patient populations (Bower, 2014; Karshikoff et al., 2017; Lasselin et al., 2017; Montoya et al., 2017) and animal models (Krzyszton et al., 2008; Mahoney et al., 2013; Bonsall et al., 2015; Norden et al., 2015; Zhang et al., 2016; Vichaya et al., 2017). Indeed, it is well-known from experimental studies that acute severe inflammation, such as induced by lipopolysaccharide (LPS), causes acute sickness behavior, including fatigue. This response has been interpreted as an adaptive process leading to the conservation of energy and reduction of the risk of further dissemination of pathogens (e.g., by withdrawing from social interactions) (Dantzer et al., 2014; Engler et al., 2016). Moreover, in autoimmune diseases such as multiple sclerosis, diabetes, and rheumatoid arthritis, the level of fatigue is associated with an increase in plasma cytokines, especially during symptom relapse (Lasselin et al., 2012; Malekzadeh et al., 2015; Patejdl et al., 2016; Choy and Calabrese, 2017). Associations between small, prolonged increases in plasma inflammatory cytokines and chemokines and persistent fatigue have also been reported in cancer survivors (Bower and Lamkin, 2013) and in individuals with chronic fatigue syndrome (Montoya et al., 2017). In these patients, the levels of plasma cytokines are generally much lower than those detected in patients with autoimmune diseases. The mechanisms by which subtle increases in inflammation induce fatigue are still unclear, and could be different from what has been shown for acute severe inflammation. However, the number of associations reported in the literature suggest that the effect is biologically significant.

**Figure 1:**
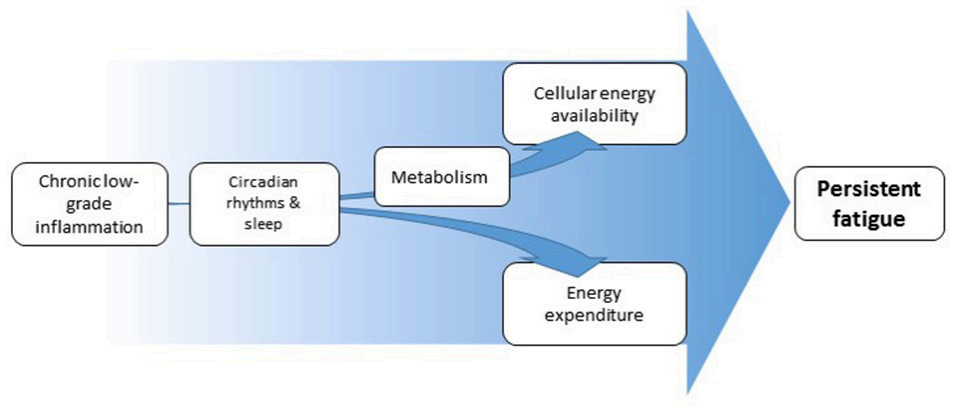
Schematic overview of proposed pathways from inflammation to fatigue. Inflammation affects cellular energy availability through its effects on metabolism. Inflammation can at the same time affect energy expenditure, both through increased energy demand by the immune system and through changes in motivation-driven energy expenditure. Both pathways can be partially mediated by altered circadian rhythms and disturbed sleep. The resulting imbalance in energy availability and expenditure underlies the experience of fatigue.

We propose that chronic low-grade inflammation induces and/or maintains persistent fatigue by inducing an imbalance between cellular-energy *availability* and cellular- and behavioral-energy *expenditure* (Figure 1). Inflammation increases the need of immune cells for rapid generation of cellular energy. To meet this need, immune cells shift to aerobic glycolysis for energy production, a less-efficient, but fast-acting pathway (Kominsky et al., 2010; McGettrick and O'Neill, 2013). During chronic low-grade inflammation, the extended reliance on aerobic glycolysis would be expected to lead to reduced nutrient availability and thus to less energy availability for demanding organ systems. The organismal energy balance can further be encumbered by changes in circadian rhythms and sleep. In addition, there is evidence suggesting that low-grade or chronic inflammation (but not acute severe inflammation) can be linked to increases in *behavioral*-energy expenditure (Vichaya et al., 2014; Lasselin et al., 2017), contributing to the imbalance between energy availability and expenditure and, thereby, leading to fatigue.

## Effects of low-grade inflammation on energy production and expenditure

**Figure 2:**
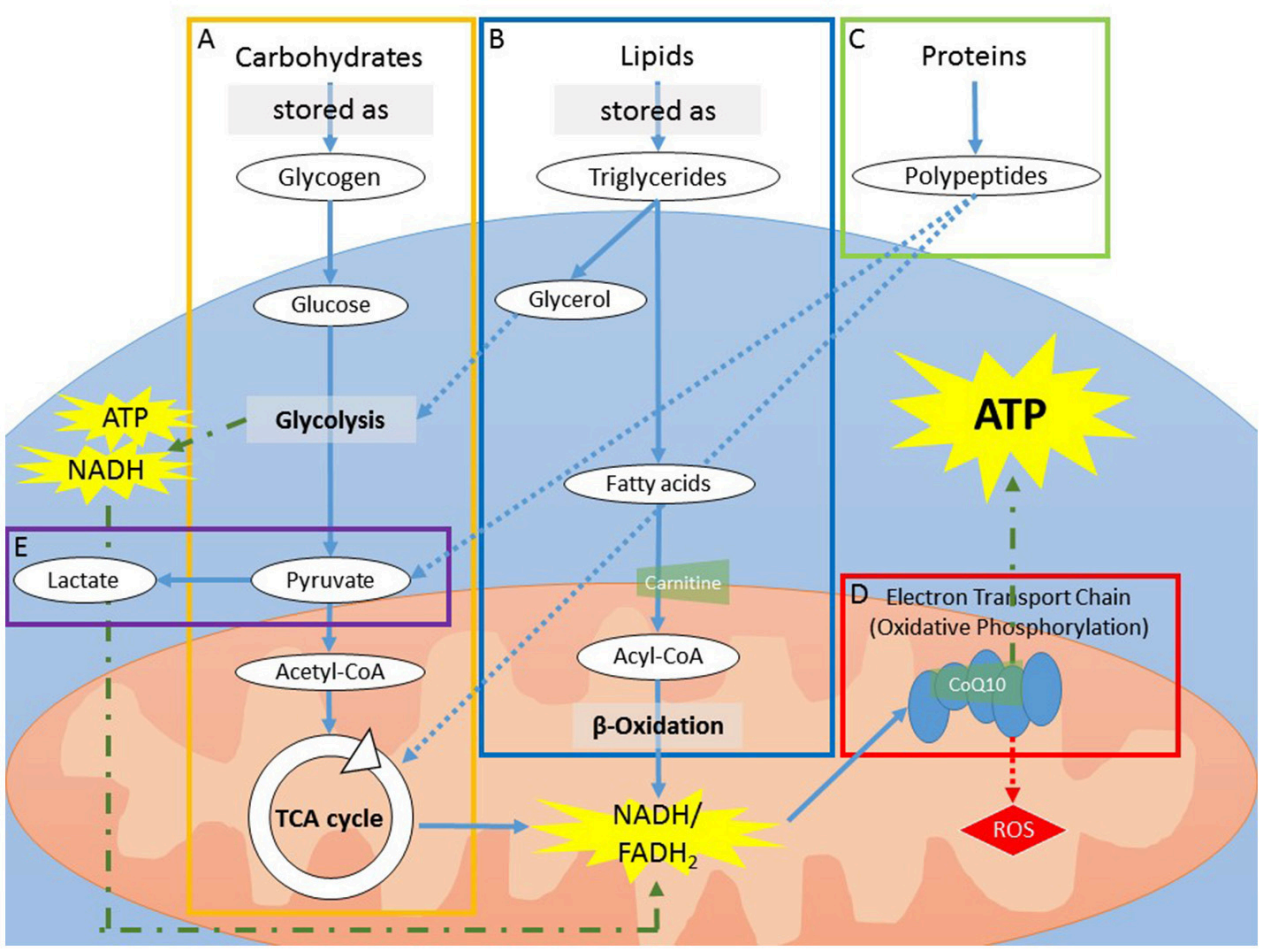
**(A)** Carbohydrates can be stored as glycogen, which can be rapidly utilized for fast energy production. As storage is limited to a handful of organs, mainly the liver and skeletal muscles, sustained usage of glycogen will deplete stores in a few h. During catabolism, glycogen is first broken down into glucose molecules that enter into glycolysis, yielding pyruvate. The process of glycolysis yields a low amount of ATP and NADH. After the addition of a CoA group, pyruvate (now acetyl-CoA) enters into the TCA cycle to produce NADH and FADH_2_. **(B)** In contrast to carbohydrate storage, storage of lipids, in the form of triglycerides, is virtually limitless. Catabolism of lipids is a slow process and is therefore mainly utilized during prolonged energy need (i.e., when carbohydrate storage is expended). The process yields fatty acids and glycerol. The addition of a CoA group to fatty acids generates acyl-CoA, which is carried into the mitochondria via the carrier protein carnitine. Once inside the mitochondria, acyl-CoA is catabolized via β-oxidation to produce NADH and FADH_2_. Glycerol can enter into the end steps of glycolysis or is reprocessed to form glucose (i.e., gluconeogenesis). **(C)** Proteins can be broken down in smaller polypeptides or amino acids during ATP production but cannot be stored. As proteins are required for biological functions other than ATP production, they are thought to be used for ATP production only in conditions of extreme demand, such as sickness or chronic inflammation. Proteins used for ATP production do not go through glycolysis, but instead are converted to TCA metabolites or pyruvate. **(D)** NADH and FADH_2_ generated by glycolysis, β-oxidation, and the TCA cycle are converted via the electron transport chain (ETC) in the mitochondria. The ETC is a series of 5 protein complexes that work in synchrony to produce ATP. This process, called oxidative phosphorylation, requires oxygen. CoQ10 functions as an electron carrier between the complexes of the electron transport chain. **(E)** In the absence of oxygen, or when mitochondria are impaired, glycolysis is the dominant energy-producing metabolic pathway. In order for glycolysis to continue, however, NAD+, a secondary substrate along with glucose, must be regenerated from NADH. To do so, pyruvate is converted into lactate, an energy-requiring mechanism that utilizes NADH and thus decreases overall ATP production. ATP, adenosine triphosphate; CoA, coenzyme A; CoQ10, coenzyme Q10; ETC, Electron transport chain; FADH_2_, flavin adenine dinucleotide; NADH and NAD+, forms of nicotinamide adenine dinucleotide; TCA, tricarboxylic acid.

Adenosine triphosphate (ATP) is the primary form of energy “currency” utilized by the cells. The generation of ATP involves the catabolism of macronutrients (carbohydrates, lipids, and proteins), each starting from a unique metabolic pathway but ultimately being shuttled to the cells for ATP production (Figure 2). Factors that can negatively affect ATP production include reduced intracellular glucose availability through either alterations in macronutrient metabolism or reduced glucose uptake by the cells; reduced functioning of the mitochondria for aerobic energy production and subsequent increased dependence on less-efficient aerobic glycolysis.

### Energy production during prolonged inflammation

**Figure 3:**
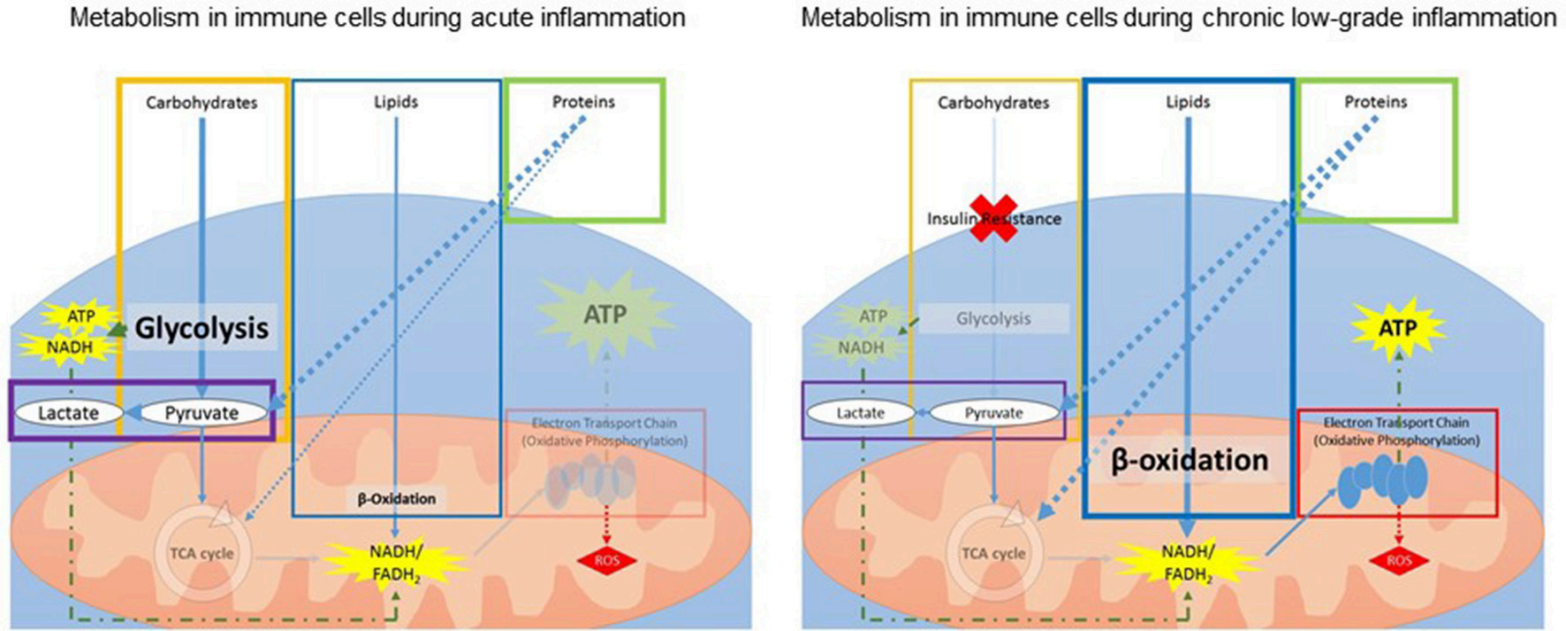
Schematic overview of changes in metabolism within immune cells during acute **(Left)** and chronic **(Right)** inflammation. During acute inflammation, immune cells increase glycolysis while decreasing the TCA cycle for fast generation of ATP. During chronic *low-grade* inflammation, insulin resistance leads to a decrease in glucose uptake and glycolysis. To compensate, the body increases lipid and protein metabolism for ATP production. ATP, adenosine triphosphate; FADH_2_, flavin adenine dinucleotide; NADH, nicotinamide adenine dinucleotide; ROS, reactive oxygen species; TCA, tricarboxylic acid.

Inflammation requires a change in metabolism, and these changes differ between acute and chronic or prolonged inflammation (Figure 3). During acute inflammation, rapid ATP production in immune cells is required for the multitude of immune responses. Immune cells (lymphocytes and leukocytes) switch from oxidative phosphorylation to an increased reliance on aerobic glycolysis for rapid ATP production (Kominsky et al., 2010; McGettrick and O'Neill, 2013; Kelly and O'Neill, 2015). This shift is in favor of precipitous ATP production while catabolic efficiency is sacrificed, resulting in decreased mitochondrial function and increased lactate production.

Additionally, prolonged inflammation is associated with increased insulin resistance and reduced glucose tolerance, leading to reduced glucose uptake by the immune cells and, consequently, less overall ATP production from glucose (Shoelson et al., 2006; Asghar and Sheikh, 2017). Therefore, during ongoing low-grade inflammation, energy and glucose are diverted from other systems to support the immune response. To compensate, the body increases lipid and protein metabolism for ATP production (Liu et al., 2012c). Lipid metabolism is a slow process and thus cannot respond to rapid energy requirements. Additionally, increasing reliance on protein metabolism means other functions, such as promotion of growth stimulation, are compromised (Le Floc'h et al., 2004). Inflammation also leads to increased reactive oxygen species (ROS) to combat invading pathogens (Liu et al., 2012a; Maitra et al., 2012). However, ROS can be harmful to mitochondria and mitochondrial DNA (Sinha et al., 2013) and thus, prolonged exposure to ROS during chronic inflammation contributes to mitochondrial dysfunction.

The effects of chronic inflammation are especially detrimental for neurons that heavily depend on oxidative phosphorylation for their ATP needs (Hall et al., 2012). In normal brain metabolism, neurons rely heavily on astrocytes. Astrocytes use glycolysis to convert lipids and glucose to pyruvate and then lactate. Lactate is then shuttled to neurons where it is enters the tricarboxylic acid (TCA) cycle. During chronic inflammation, insulin resistance decreases astrocytic glucose and protein metabolism, resulting in reduced lactate availability for neurons (Blázquez et al., 2014), forcing the neurons to rely more heavily on the slower process of lipid metabolism. Further, inflammation is able to low mitochondrial efficiency (Yan et al., 2013), increasing the metabolic burden on neurons. Alterations in neuronal metabolism will ultimately affect neuronal functioning.

### Behavioral-energy expenditure during inflammation

Chronic low-grade inflammation has been estimated to increase systemic energy expenditure by up to 10% (Straub, 2017). Given the increased energy demand of chronic inflammation, and the resulting reduced energy availability, one would expect that *behavioral*-energy expenditure (i.e., amount of energy spent on activities) would decrease. Several studies in the context of acute, severe inflammation corroborate this hypothesis showing reductions in willingness (motivation) to exert effort, both in rodent models (Larson et al., 2002; Felger et al., 2013; Nunes et al., 2014; Yohn et al., 2016) and in humans (Draper et al., 2017) (Table 1). However, results from these and other studies also suggest that motivational behavior might be differentially affected in conditions of low-grade inflammation.

**Table 1:** Overview of discussed studies on the effects of inflammation on effort expenditure.

**References**	**Study description**	**Study targets**	**Main results on motivational effort expenditure**
**ANIMAL MODELS**			
Larson et al., 2002	Mice tested in an operant conditioning paradigm 90 min after IL-1β or saline control. Testing included willingness to perform nose pokes for a reward (sweetened milk) under fixed ration schedules (FR; fixed number of nose pokes needed for reward) and progressive ratio schedules (PR; number of required nose pokes increases incrementally with each reward).	Comparison of saline vs. IL-1β (30, 100, and 300 ng). Outcome was number of completed trials within a given time (response rate) on FR4, 10, or 32 or maximal number of responses made for a single pellet (breaking point) on a PR10 schedule.	100 and 300 ng IL-1β led to decreased response rate on the FR32 and to a significant decrease in the breaking point on the PR10. *30 ng led to slight (nonsignificant) increases in response rate in the FR10 and in break point in the PR10, suggestive of increased willingness to exert effort under low-grade inflammation*.
Felger et al., 2013	Rhesus monkeys were tested after 4 weeks of saline vs. IFN-α treatment (administered 5 days per week to mimic monotherapy for malignant melanoma) in a randomized repeated measures design.	Comparison of saline vs. IFN-α treatment on willingness to work for a sucrose treat. Outcome was the number of sucrose pellets obtained from a puzzle feeder (requiring work to obtain the treat).	IFN-α treatment led to a reduction in sucrose pellets obtained from the puzzle feeder. Consumption of pellets from the regular feeder was not reduced by IFN-α, suggesting no change in anorexia or appetitive behavior.
Nunes et al., 2014	Rats were tested in an FR5-lever pressing protocol 90 min after saline or IL-1β in the presence of freely available but less preferable regular chow.	Saline vs. IL-1β (1.0, 2.0, and 4.0 mg/kg). Outcomes were response rates on the FR5 and amount of freely available chow consumed.	IL-1β at 2.0 and 4.0 mg/kg dose decreased response rates compared to saline. This was paired with significant increases in consumption of freely available regular chow, suggesting that the effects of IL-1β were not due to change in appetitive behavior. No effect was observed for the 1.0 mg/kg dose of IL-1β.
Yohn et al., 2016	Rats were tested in an FR5-lever pressing protocol 45 min after saline or IL-6 administration in the presence of freely available but less preferable regular chow.	Saline vs. IL-6 (2.0, 4.0, 6.0, and 8.0 mg/kg). Outcomes were response rate on the FR5 and amount of freely available chow consumed.	IL-6 at 4.0–8.0 mg/kg dose led to decreased response rate compared to saline. Microdialysis revealed reduced extracellular dopamine in the accumbens following IL-6 administration.
Vichaya et al., 2014	Mice tested approximately 24 h after LPS or saline on a concurrent choice operant conditioning task (FR-10 for preferred chocolate pellets and FR-1 for a less-preferred grain pellet).	Saline vs. LPS (0.33 mg/kg). Outcome was total number or nose pokes for chocolate and grain rewards as well as chocolate preference (% chocolate pellets earned).	While LPS led to a reduction in total number of nose pokes, this shift was mostly driven by a reduction in nose pokes for grain, resulting in an increase in percentage of chocolate pellets after LPS.
**CLINICAL STUDIES**			
Lasselin et al., 2017	Healthy subjects (*n* = 21; 9 women) tested on effort expenditure 5 h after LPS vs. saline injection in a cross-over design.	LPS (2 ng/kg) vs. saline. Outcome was ratio of high effort-high reward/low effort-low reward choices under different reward conditions.	Subjects showed an increase in ratio of high effort choices after LPS, only when reward conditions were more preferable. The effect of LPS was mediated by increased sleepiness. *Subjects were tested 5 h after injection at which time plasma IL-6 concentrations and acute sickness symptoms had started to decrease in the LPS condition*.
Draper et al., 2017	Healthy subjects (*n* = 29; all male) tested on effort expenditure choices 2 and 5 h after LPS vs. saline injection in a cross-over design.	LPS (2 ng/kg) vs. saline. Outcome was number of accepted offers to exert effort (squeezing a dynamometer) for a reward under different reward conditions.	Subjects accepted fewer offers with high effort requests 2 h after LPS. No effect of LPS was found after 5 h. *At 2 h post injection IL-6 and TNF-α concentration were at their peak, but sickness symptoms were decreased. No effects on effort expenditure were found 5 h post LPS, at which time IL-6 and TNF-a levels had almost returned to control levels*.

Studies on animal models show a reduction in effort expenditure for food only after administration of higher doses of inflammatory mediators (Larson et al., 2002; Felger et al., 2013; Nunes et al., 2014; Yohn et al., 2016). For the lowest doses, not only was there no reduction in activity, there also seemed to be a trend for increased activity (Larson et al., 2002). Further, Vichaya et al. (2014) tested animals 24 h after an acute inflammatory insult (LPS injection), at which point the acute inflammatory response and sickness behavior had subsided. A shift was observed in motivational priority *toward* choices that required a higher effort for a more salient reward.

Comparing the results from the two available studies in humans also point to distinct effects of high vs. low-grade inflammation on effort expenditure choices. Draper et al. (2017) tested healthy individuals 2 and 5 h after LPS/saline injection and found a reduced willingness to perform high-effort tasks in LPS-treated participants at 2, but not 5, h post injection. At 2 h, inflammation was as its peak. In contrast, Lasselin et al. (2017) tested participants 5 h post LPS/saline injection and observed an *increased* willingness to perform high effort tasks in LPS-treated participants (but only when the conditions to win were favorable). Lasselin et al. further noted that participants were too weak to be tested between 3 and 4 h post injection. Of note, the two studies differed in several important aspects including sample characteristics (only males vs. mix of males and females) and task design (choice between accepting or rejecting a task vs. choice between a high effort/high reward or a low effort/low reward task) (see Table 1 for study specifics). Nevertheless, we hypothesize on the basis of these human and murine experimental data that acute severe inflammation leads to overall adaptive *reductions* in effort expenditure, whereas mild inflammation can lead to relative *increases* in effort expenditure provided the incentive is strong enough. This notion is supported by our findings that low-grade inflammation was associated with *increased* high-effort choices in a sample of cancer patients and survivors (Lacourt et al., under review). It is still to be determined if these behavioral shifts in effort expenditure correspond to “recovery” or to a switch in immune cells back from a glycolytic to a more oxidative-driven metabolic profile. In addition, behavioral shifts during *chronic* low-grade inflammation have not yet been studied.

The effects of inflammation on effort expenditure in motivational tasks appear to be mediated by the dopaminergic system (Felger et al., 2013; Yohn et al., 2016). Although the mechanism by which inflammation is capable of altering dopamine neurotransmission is still unclear, the literature on Parkinson's disease indicates that chronic neuroinflammation leads to oxidative stress and mitochondrial damage in dopaminergic neurons (Niranjan, 2014; Blesa et al., 2015), leading to an impairment in dopaminergic neurotransmission. Dopaminergic neurons are particularly vulnerable to inflammation (de Pablos et al., 2014; Park et al., 2016). Although the cause of this vulnerability is not fully understood, the higher basal oxidative phosphorylation of these cells and the auto-oxidation of excess extracellular dopamine may contribute (Asanuma et al., 2003; Pacelli et al., 2015).

### Sleep and circadian rhythms as partial mediators of the effects of chronic low-grade inflammation on energy availability and expenditure

Inflammation can additionally lead to more-sustained alterations in metabolism via changes in circadian rhythms and sleep. Chronic low-grade and severe inflammation has been shown to affect sleep quality and circadian rhythms (Pollmächer et al., 2000; Haspel et al., 2014; Anderson et al., 2015), and vice versa (Leproult et al., 2014; Rahman et al., 2015; Wright et al., 2015). Alterations in circadian activity rhythms have been linked to fatigue (Payne, 2011; McHill and Wright, 2017). Sleep disturbances or reduced sleep quality—often reported by individuals with persistent fatigue—can also lead to disorganized or misaligned circadian rhythm through daytime napping and structural alterations in nighttime sleep onset.

Most metabolic processes are regulated by the circadian clock, and changes in circadian rhythm or sleep are associated with metabolic changes, such as increased circulating glucose and decreased insulin sensitivity (Depner et al., 2014; Potter et al., 2016). Specifically, expression of mitochondrial proteins involved in many metabolic processes undergo posttranslational modifications regulated by the *Clock* gene (Masri et al., 2013). In addition, mitochondrial oxidative metabolism is also controlled by the circadian clock through oscillations in biosynthesis of nicotinamide adenine dinucleotide (NAD+) and availability of rate-limiting mitochondrial enzymes (Peek et al., 2013; Neufeld-Cohen et al., 2016).

Insufficient sleep and changes in circadian rhythms can also alter cellular-energy and behavioral-energy expenditure. Preclinical studies show increased energy expenditure after total sleep deprivation in rats (Caron and Stephenson, 2010). Likewise, experimentally induced sleep deprivation in healthy human volunteers led to increased daily energy expenditure, assessed via whole-room calorimetry (oxygen consumption and carbon dioxide production). Although this was accompanied by a compensatory increase in energy (food) intake (i.e., caloric intake exceeded caloric expenditure), physical exhaustion increased significantly (Markwald et al., 2013). As the above described studies rely on experimentally induced alterations in circadian rhythm and sleep, it is unclear to what extent these findings are relevant for naturally occurring changes. In addition, whether more subtle, sustained changes in sleep and circadian rhythm, such as observed in fatigued individuals, also alter energy expenditure remains to be shown.

### Summary of proposed pathways

Chronic low-grade inflammation is related to a persistent metabolic switch to faster, but less efficient, glycolytic energy production, and increased ROS production. This in its turn affects mitochondrial function, thereby increasing the reliance on glycolytic pathways. In addition, although chronic inflammatory processes demand more energy, this increased need for energy is not always accompanied by decreases in behavioral-energy expenditure. This seems particularly the case in the context of low-grade inflammation, which has been shown to increase motivation for effort expenditure. Inflammation can also affect circadian rhythms and sleep (and vice versa), which can further exacerbate the effects on energy availability and expenditure.

We propose that this imbalance of energy availability vs. expenditure underlies the experience of fatigue induced by chronic low-grade inflammation. Below, we discuss the evidence for these metabolic pathways in persistent fatigue.

## Persistent fatigue as a result of reduced energy availability and increased energy expenditure?

Associations between low-grade inflammation and persistent fatigue have been reported predominantly for individuals with CFS or CRF. In patients with CFS, fatigue severity has been associated with elevated cytokine concentrations in plasma (e.g., Montoya et al., 2017) and spinal fluid samples (e.g., Hornig et al., 2016). Alterations in physiological stress-response (predominantly hypothalamic-pituitary-adrenal axis output) in patients with CFS have also been reported (e.g., Silverman et al., 2010), suggesting that low-grade inflammation might be the result of reduced sensitivity to immune-regulating stress hormones such as cortisol. For CRF, fatigue usually starts during cancer treatment but persists into survivorship in a significant subset of patients (Servaes et al., 2002; Goedendorp et al., 2013). Most first-line treatments such as chemotherapy and surgery are known to induce an inflammatory response (e.g., due to tissue damage) (Fitzpatrick and Wheeler, 2003; Boomsma et al., 2010; Wang et al., 2016). Increases in inflammatory markers have indeed been associated with increased fatigue severity in patients actively undergoing treatment, although not all inflammatory markers associated with fatigue show a response to cancer treatment, suggesting the presence of other causal factors (Reed et al., 2016). In cancer survivors, despite the cessation of the immediate inflammatory effects of cancer treatment, associations between elevated levels of inflammatory markers and increased fatigue have also been observed (Bower, 2007). CRF in survivors has in addition been associated with psychosocial stress factors, such as fear of cancer recurrence (Young and White, 2006), suggesting that inflammatory processes during survivorship might (in part) be stress-related (Lacourt and Heijnen, 2017).

### Reduced energy availability in persistent fatigue

Evidence of reduced energy availability in persistent fatigue comes mainly from metabolomic studies (e.g., Fluge et al., 2016; Naviaux et al., 2016; Yamano et al., 2016) and from studies of mitochondrial dysfunction (e.g., Filler et al., 2014).

#### Mitochondrial dysfunction

Mitochondria are often represented as the “powerhouses of the cell.” Mitochondrial function can be compromised by a multitude of factors, including reduced availability of necessary metabolites and mitochondrial damage through, for example, oxidative stress (Box 1). The notion that mitochondrial dysfunction can lead to fatigue is indicated by the observation that fatigue is a common symptom of mitochondrial disease (Gorman et al., 2015) and that fatigue-like behavior in animal models is associated with reduced expression of mitochondrial complexes, reductions in metabolic activity, and alterations in mitochondrial morphology in the brain (Surapaneni et al., 2012; Wang et al., 2014; Vichaya et al., 2016).

Box 1 **Mitochondria and biomarkers of mitochondrial function.**

Healthy mitochondrial functioning depends on a multitude of factors. Mitochondria themselves produce reactive oxygen species (ROS) as a byproduct of metabolism. In normal conditions, antioxidants are sufficient at protecting against damage from ROS, and the cell remains in equilibrium. However, if the balance skews toward ROS production during excessive and/or dysfunctional mitochondrial activity, ROS can induce the production of several toxins and can damage the cell; this is referred to as oxidative stress.

Most patient studies on biomarkers of mitochondrial function have focused on circulating levels of coenzyme Q10 (CoQ10) or *L-*carnitine. **CoQ10** is a component of the electron transport chain of the mitochondria and is thus important for mitochondrial energy production. It is assumed that CoQ10 serum levels mirror levels in the mitochondria, although to the best or our knowledge this has not been formally studied. The antioxidant properties of CoQ10 make it an anti-inflammatory agent as well as an important mitochondrial protectant (Genova et al., 2003; Sanoobar et al., 2015). *In vitro* and animal studies have indeed shown a beneficial effect of CoQ10 on mitochondria (Bergamini et al., 2012; Jiménez-Santos et al., 2014). ***L-*****carnitine** can be either made by the body or acquired in the diet. L-carnitine is important for transport of fatty acids across the mitochondrial membrane for β-oxidation and adenosine triphosphate production. Thus, reductions in L-carnitine indicate reduced availability of fatty acids in the mitochondria and reduced tricarboxylic acid activity (Porter et al., 2017).

The association between mitochondrial dysfunction and persistent fatigue has been most widely studied in patients with CFS (Table 2). A systematic review reported that 21 of 25 papers were restricted to CFS (Filler et al., 2014). Lower serum levels of the antioxidant CoQ10 was the most consistent finding of the review, with limited evidence for lower levels of other antioxidants and increased levels of oxidative stress markers. Lower carnitine levels were observed in some studies, but results depended on the type of carnitine assessed. Later published studies on CFS confirm increased oxidative stress levels, possible decreases in ATP production efficiency, and reductions in mitochondrial energy production in patients with CFS (Ciregia et al., 2016; Tomas et al., 2017). The assessment of oxygen consumption in PBMCs by Tomas et al. (2017) is promising, indicating a reduced capacity of mitochondria to meet increased energy demands during cellular stress, a replication is warranted based on the use of both frozen and fresh blood samples and the limited sample size.

**Table 2:** Overview of discussed studies on the association between fatigue and cellular energy production.

**References**	**Study design and sample description**	**Description**	**Relevant results**
**MITOCHONDRIAL FUNCTIONING—OBSERVATIONAL STUDIES**			
**CFS**			
Filler et al., 2014	Review; 25 papers of which 20 included patients with CFS/ME, which are summarized here.	Description of studies assessing associations between fatigue and outcomes of mitochondrial function.	Most consistent evidence for lower serum levels of CoQ10 in patients with CFS (4/4 studies). Other promising findings included reduced carnitine levels (4/5 studies); decreased antioxidant levels (2/2 studies); changes in mitochondrial structure (3/4 studies); and impaired energy production (2/4 studies).
Ciregia et al., 2016	Cross-sectional study; Twins discordant for CFS; Further validation in a sample of patients with CFS (*n* = 45) and healthy controls (*n* = 45).	Proteomic analysis of platelet-derived mitochondria.	Initial and validation analyses showed upregulation in aconitate hydratease (ACON) and ATP synthase subunit beta (ATPB). *ACON is a biomarker for increased oxidative stress and ATPB is associated with ATP production. The authors suggest that the observed upregulation in ATPB indicates an attempt to increase ATP production due to ATP production inefficiency*.
Tomas et al., 2017	Cross-sectional study; Patients with CFS (*n* = 63) and controls (*n* = 15).	Oxygen consumption and glycolytic activity in PBMCs (fresh or frozen).	Samples from CFS patients showed reductions in basal respiration, proton leak, maximal respiration, and spare capacity. No differences were found in glycolytic activity. *Fresh samples were available for only 3 controls and strong differences were observed between fresh and frozen samples, reducing the reliability of the findings. Strongest effect was found for reductions in maximum capacity*.
**CRF**			
Hsiao et al., 2013	Longitudinal observational study; Non-metastatic prostate cancer patients (NMPC) (*n* = 15); Healthy controls (*n* = 15) were included for reference values.	Change in mitochondria-related gene expression in peripheral blood samples in association with change in fatigue during external beam radiation therapy (EBRT).	Gene expression and fatigue severity did not differ at baseline between patients and controls. In patients, fatigue increased during EBRT. Of the 11 genes that were differentially expressed during EBRT (as compared to baseline), 8 were significantly associated with fatigue scores during radiation. Upregulated genes: BCL-2, FIS1, SLC25A37; downregulated genes: AIFM2, IMMP2L, MSTO1, SLC25A23, and SLC25A24.
Hsiao et al., 2014	Longitudinal observational study; NMPC patients (*n* = 25) undergoing (+EBRT); NMPC patients on active surveillance (*n* = 25) were included as controls (−EBRT).	Changes in expression of 168 mitochondria-related genes in peripheral blood samples in association with fatigue during EBRT.	Patients +EBRT and -EBRT did not differ in fatigue severity or gene expression at baseline. Patients +EBRT showed increased fatigue during treatment. Out of 14 genes that were differentially expressed during EBRT (as compared to baseline), 4 genes were associated with fatigue severity at baseline and during EBRT. Increased fatigue – downregulation of gene: BCL2LI, SLC25A37, FIS1; increased fatigue – upregulation of gene: BCS1L. Confirmatory protein expression analyses showed no associations between fatigue scores and gene-related protein concentrations.
Lukkahatai et al., 2014	Longitudinal observational study; NMPC patients undergoing EBRT (*n* = 12).	Serum proteomic profile before and midway through EBRT (day 21).	Apolipoprotein A1 (Apo1), ApoE, and transthyretin (TTR) were identified to have changed between baseline and day 21. Patients were post hoc divided into high fatigue (*n* = 9; higher fatigue during EBRT) and no fatigue (*n* = 3) based on their fatigue at day 21. The group characterized as ‘high fatigue’ had higher ApoA1 expression at day 21 but not at baseline and showed an increase in ApoE during treatment, whereas the ‘low fatigue’ group did not. TTR values did not differ between groups and did not change significantly in either group.
Filler et al., 2016	Longitudinal observational study; NMPC patients (*n* = 22) undergoing EBRT.	Expression of enzymes of mitochondrial oxidative phosphorylation complexes (complexes I-V) and the antioxidant Manganese superoxide dismutase (MnSOD) in serum in association with changes in fatigue between pre-EBRT and the last day of EBRT.	Lower expression of Complex II enzymes were associated with decreased fatigue scores at baseline and at completion of EBRT. *Post-hoc* characterization of patients as high fatigue and low fatigue showed that the between complex II enzymes and fatigue was only observed in the ‘high fatigue’ group. Enzyme levels for every complex showed an increase in the high fatigue group and a decrease in the low fatigue group; these within-subgroup changes were not significant. MnSOD did not change significantly in either group.
**EXPERIMENTAL STUDIES AND CLINICAL TRIALS**			
**CFS**			
Brown et al., 2015	*In vitro* study of skeletal muscle cells cultures; Cells obtained from patients with CFS (*n* = 10) and age-matched controls (*n* = 7).	Effects of electrical pulse stimulation (EPS) of skeletal muscle cells.	EPS led to insulin-stimulated glucose uptake in control samples but not in CFS samples. EPS-induced IL-6 secretion was seen in samples from both groups but overall IL-6 secretion was lower in the CFS samples. Both groups showed a similar increase in lactate dehydrogenase in response to EPS.
Snell et al., 2013	Experimental study; Patients with CFS (*n* = 51) and controls (*n* = 10).	Physiological responses to repeated maximal exercise tests	Patients with CFS reached their ventilator threshold (VT) at a lower workload during the second exercise test while controls did not show a change in workload at VT.
Castro-Marrero et al., 2014	Randomized controlled clinical trial; Patients with CFS (*n* = 73).	Effects of 8-week oral CoQ10 and NADH supplementation vs. placebo on fatigue and metabolic outcomes.	Supplementation led to a reduction in fatigue and increased PBMC levels of NAD+, ATP, CoQ10, and citrate synthase activity as well as lower NADH and lipid peroxidation. No changes were observed in the placebo group.
**CRF**			
MacCiò et al., 2012	Randomized controlled clinical trial; Advanced-stage gynecological cancer patients with cachexia (*n* = 124).	Evaluating the effects of 4-month treatment with either synthetic progestogen alone (standard cachexia treatment) or with addition of L-carnitine, celecoxib, and antioxidants.	Additional supplementation led to stronger decreases in fatigue, resting-state energy expenditure (indirect calorimeter), IL-6 and TNF-a concentrations, and ROS.
Iwase et al., 2016	Open label clinical trial; Breast cancer patients undergoing chemotherapy.	Effects of 21-day supplementation with amino-acid jelly containing CoQ10 and L-carnitine or standard-of-care on reported fatigue.	Patients reported less-severe fatigue after supplementation.
Lesser et al., 2013	Randomized controlled clinical trial; Breast cancer patients planned for adjuvant chemotherapy (*n* = 236).	Effects of 24-weeks supplementation with vitamin E ± CoQ1 on reported fatigue.	Supplementation increased plasma CoQ10 levels, but did not affect fatigue outcomes.
**ANIMAL MODELS**			
Davis et al., 2009	Mice were required to run to exhaustion on a treadmill or were provided access to a voluntary wheel.	Evaluated effect of 12.5 or 25 mg/kg quercetin (antioxidant/ anti-inflammatory) via oral gavage for 7 days prior to treadmill test. For voluntary wheel running mice were supplemented in their food.	Both doses of quercetin increased maximal endurance capacity and the 25 mg/kg dose increased voluntary wheel running activity. Further, both doses increased PGC1α, SIRT1, and cytochromie c in the brain and soleus muscle. Only the 25 mg/kg dose increased brain and soleus mtDNA copy number.
Fu et al., 2010	Mice were subjected to weight-loaded forced swim for 30 min after the final drug treatment.	Evaluated effect of CoQ10 (0, 1.5, 15, or 45 mg/kg/day for 4 weeks) on fatigue-like behavior.	The 15 mg/kg/day dose of CoQ10 increased swim time to exhaustion. CoQ10 also decreased urea nitrogen post-exercise, increased pre-exercise glycogen (at 15 and 45 mg/kg doses), and had no significant impact on lactic acid.
Singh et al., 2002a	Mice subjected to forced swim (6 min/day for 7 days) as a model of CFS	Evaluated effects of concurrent administration of various agents [i.e., (alleged) anti-oxidants GS-02, melatonin, carvedilol, and St. Johns wort; antidepressant fluoxetine].	Carvedilol, melatonin, St. Johns wort, and GS-02 all reduced immobility from days 2 to 7. Fluoxetine reduced immobility in the swim test on days 1–2, but had no effect on days 3–7. Further, antioxidant treatment, but not fluoxetine, reduced brain enzyme levels of MDA and catalase while increasing GSH and SOD levels.
Singh et al., 2002b	Mice subjected to forced swim (6 min/day for 15 days) as a model of CFS	Evaluated antioxidant effects of various agents [i.e., (alleged) antioxidants withania somnifera root extract, quercetine, melatonin, carvedilol, and St. Johns wort].	As described above, the authors report beneficial effects on immobility time from melatonin, carvedilol, and St Johns wort. Quercetine and withania somnifera also showed protective effects, with the least immobility shown in the withania somifera group. All groups showed reductions in brain MDA.
Surapaneni et al., 2012	Forced swim (15 min/day for 21 days) as a model of CFS in rats	Evaluated behavior and measures of mitochondria function in control mice and those supplemented during the 21 days with withania somnifera and shilajit.	Forced swimming increased immobility during swimming, enhanced anxiety-like behavior, reduced mitochondrial membrane potential, reduced mitochondrial parameters (e.g., NADH, SDH, Cyto c oxidase, ATP synthase). These effects were attenuated by withania somnifera and shitajit.
Vichaya et al., 2016	Mouse model of cancer and cancer-therapy (cisplatin + leg radiation)	Described cancer and therapy induced behavioral changes (burrowing) and brain and liver mitochondria complex gene expression.	The most profound effect on behavior and brain mitochondria complex gene expression was in the tumor-bearing mice treated with cancer therapy. (Note that liver mitochondrial complex gene expression was most effected in the tumor-untreated group.)
Wang et al., 2014	Forced swim (6 min/day for 15 days) as a model of CFS in mice	Evaluated the antioxidant effects of polysaccharides from Panax ginseng (WGPA-A, WGPA-N).	WGPA-A, but not WGPA-N, prevented swim induced enhanced immobility, reduced serum markers of oxidative stress, and protected against ultra-structural changes of striated muscle mitochondria.
Zhuang et al., 2014	Rats were tested in a model of post-operative fatigue (70% removal of small intestine) using open field activity	Evaluated the antioxidant effect of 15 mg/kg/day Ginsenoside Rb (GRb1) starting 3 days prior to surgery.	On day 1 and 3 post surgery, rats showed reduced activity compared to controls, this reduction was not observed in the GRb1 treated rats. Skeletal muscle SOD, Nrf2, and Akt levels were increased by surgery and further increased by GRb1. Surgery also increased muscle MDA and ROS and these effects were attenuated by GRb1.
**METABOLOMIC STUDIES**			
**CFS**			
Yamano et al., 2016	Metabolomics analyses of plasma samples; Samples from patients with CFS (*n* = 46) and healthy controls (*n* = 47); Further validation in a new sample of 20 CFS patients (*n* = 20) and healthy controls (*n* = 20).	Metabolites were identified with capillary electrophoresis time-of-flight mass spectrometry. Out of 144 metabolites identified, 31 with large signal/noise ratio and few missing data were observed in both data sets and thus used for group comparisons.	Initial and validation analyses showed a reduction in the ratio of pyruvate/isotrate and of ornithine/citrulline. Pyruvate was increased and isotrate was decreased in samples from CFS patients, suggesting a reduction in TCA cycle activity, possibly due to a disturbed link between glycolysis and the TCA cycle. Ornithine was higher and citrulline was lower in samples from CFS patients, suggesting a reduction in activity of the urea cycle at entry point of the cycle. Interestingly, these steps take place in the mitochondria, while subsequent steps take place in the cytosol.
Naviaux et al., 2016	Metabolomics analyses of plasma samples from patients with CFS (*n* = 45) and healthy controls (39).	Metabolites were identified with targeted, broad-spectrum, chemometric analysis. Out of 612 metabolites assessed, 420 metabolites that could be identified in all samples and were used for analyses.	The most dominant metabolic disturbances identified in both male and female patients with CFS pertained to sphingolipid pathways, driven by a decrease in plasma sphingolipids and glycosphingolipids. These metabolites also correlated with performance status, but associations with fatigue severity were not reported. *Note, lipids have a broad range of action making interpretation of the findings by Naviaux difficult. It has been suggested that the observed reduced levels could be due to reduced physical activity in CFS patients (Roerink et al., 2017). Physical activity levels were not assessed in the study*.
Fluge et al., 2016	Metabolomics analyses of plasma samples from patients with CFS (*n* = 200) and healthy controls (*n =* 102).	Assessed 20 standard amino acids using gas or liquid chromatography-tandem mass spectrometry.	Amino acids that are converted to acetyl-CoA for entry in the TCA cycle were reduced in CFS samples. In addition, amino acids that are converted to TCA cycle intermediates are also reduced, more dominantly so in females with CFS. *Note, the identified amino acids were not associated with fatigue severity, but with BMI and age*.
**ANIMAL MODELS**			
Ma et al., 2015	Metabolomics analyses of urine from mice treated or not with salidroside (to alleviate fatigue) subjected to a forced swim test.	Liquid chromatography coupled mass spectrometry was performed to identify an “anti-fatigue” profile.	Several metabolites were upregulated by salidroside, such as: geranyl diphosphate (indirectly regulates lipid synthesis and protein degradation), sebacic acid (a product of fatty acid metabolism), and N-acetylserotonin (antioxidant). Salidroside was associated with a down regulation of metabolites, such as: taurine (sulfur amino acid with many biological functions), sorbitol (involved in glucose metabolism), and sebacic acid (can be oxidized to acetyl-CoA and succinyl-CoA).

Studies on CRF have thus far been limited to the immediate effects of cancer treatment, and it is unclear to what extent these effects are still present into survivorship. Preclinical research has shown that both cancer and cancer therapy are associated with mitochondrial dysfunction (Dumas et al., 2011; Tzika et al., 2013; Luo et al., 2014; Gouspillou et al., 2015; Sridharan et al., 2015; Tabassum et al., 2015; Chiu et al., 2016; Gilliam et al., 2016; Vichaya et al., 2016). It is unknown from preclinical models whether treatment-induced or cancer-induced changes in mitochondrial function are associated with fatigue. However, a handful of observational patient studies from one research group report an association in prostate cancer patients undergoing radiation therapy. Their results point to reduced mitochondrial functioning in association with fatigue, as evidenced by reduced expression of mitochondria-related genes (Hsiao et al., 2013, 2014), decreased expression of mitochondrial electron transport complex II enzymes (Filler et al., 2016), and increased apoliprotein A1 (ApoA1) in association with higher fatigue (Lukkahatai et al., 2014). ApoA1 is important for lipid scavenging, and an increase in ApoA1 is indicative of a reduced use of lipids for ATP synthesis.

A handful of studies and clinical trials corroborate the above-described observational findings. Two experimental studies in patients with CFS showed alterations in metabolic processes. Results of *in vitro* stimulation of skeletal muscle cells from patients with CFS were indicative of insulin resistance or decreased sensitivity of insulin receptors (Brown et al., 2015). Further, patients with CFS had a slower recovery from an initial maximal exercise test, leading to a more rapid reliance on less-efficient glycolytic metabolism during a subsequent test (Snell et al., 2013).

Antioxidant supplementation can be effective in reducing fatigue, suggesting a causal relationship between reduced availability of antioxidants and fatigue. In patients with CFS, supplementation with CoQ10 and nicotinamide adenine dinucleotide (NADH) reduced fatigue, whereas a placebo treatment did not (Castro-Marrero et al., 2014). Supplementation has also been shown to improve CRF. Cachexic patients with advanced-stage gynecological cancer seemed to benefit from the addition of several metabolism-related supplements to their standard cachexia treatment with synthetic progestogen, showing decreases in fatigue, inflammation, and resting-state energy expenditure as compared to standard treatment alone (MacCiò et al., 2012). In breast cancer patients undergoing chemotherapy, supplementation with an amino-acid jelly that contained CoQ10 and L-carnitine led to less-severe fatigue (Iwase et al., 2016). As this study included a standard-of-care condition as control, a placebo effect cannot be ruled out. In contrast, a large study of breast cancer patients undergoing chemotherapy using supplementation of vitamin E with or without CoQ10 did not find any effect of the added CoQ10 on fatigue development (Lesser et al., 2013). In animal models, it has also been shown that various agents with antioxidant properties can reduce fatigue-like behavior (Singh et al., 2002a,b Davis et al., 2009; Fu et al., 2010; Zhuang et al., 2014). For these studies, it should be noted that the models of fatigue pose a significant limitation. As the mechanisms underlying CFS are yet ill-defined, the validity of animal models of CFS are questionable. Further, many of these studies induce and quantify fatigue in the same intervention (e.g., forced swim), which is inherently problematic. Therefore, much work is needed to improve our models systems and validate these findings.

In sum, most evidence for an association between fatigue and mitochondrial functioning comes from CFS, indicating lower levels of antioxidants and possible reductions in mitochondrial ATP production. While lower antioxidant levels were not found in cancer patients (Filler et al., 2016), alterations in mitochondrial gene expression do indicate a role for mitochondrial functioning in fatigue. Results from clinical and pre-clinical studies point toward possible beneficial effects of mitochondria-supporting supplements. However, the majority of observational and intervention studies suffer from severe limitations in either sample size or study design. In addition, the exact causes for reduced antioxidant levels or ATP production have not been studied. While inflammation is a likely cause, it is definitely not the only candidate. Thus, additional preclinical research to identify mechanisms, as well as clinical replications in larger samples using placebo controlled-designs and relevant biomarkers as output, are urgently needed.

#### Metabolomics

Metabolomic studies allow for a broad assessment of alterations in metabolism (Table 1). Three such studies have been reported in CFS, each with different results, but all pointing toward reduced metabolic activity. Yamano et al. showed evidence for decreased activity in the TCA cycle and the urea cycle (Yamano et al., 2016). Naviaux reported downregulated metabolites of two classes of lipids in their CFS group (Naviaux et al., 2016) and Fluge reported reduced concentrations of the amino acids that act as precursors for acetyl-Coenzyme A, one of the primary inputs of the TCA cycle, in CFS patients (Fluge et al., 2016). While Yamano et al. and Naviaux et al. did not report on associations between metabolites and fatigue severity, Fluge et al. reported that there were no associations. Rather, they observed associations with age and body mass index, suggesting that their findings were not specific for fatigue in the CFS patients.

There has been limited work on metabolomics in animal models of fatigue; however, in one study of exercise-induced fatigue, several potential “antifatigue” metabolomic biomarkers were identified (Ma et al., 2015). One was geranyl diphosphate, which can indirectly regulate lipid synthesis and protein degradation. Sebacic acid, a consequence of fatty acid metabolism that can be oxidized into metabolic intermediates for the TCA cycle, was another.

In summary, results from the three metabolomic studies point to alterations in lipid and fatty acid metabolism and decreased TCA activity in relation to fatigue, which resemble metabolic changes during chronic low-grade inflammation (Figure 3). As the TCA cycle is needed to create the precursors for the oxidative phosphorylation process, decreased activity would indicate reduced ATP production via reduced oxidative phosphorylation. As lipid metabolism, or lipolysis, generates the most energy per gram of substrate, it is the most efficient manner of energy storage. As noted in Figure 2, the catabolism of lipids to usable energy is a slow process and mainly utilized during extended energy need, such as chronic inflammation. Thus, alterations in this metabolic pathway might result in reduced availability of efficient energy sources, creating an increased reliance on carbohydrate-based metabolism. In addition, reduced fatty acid availability would interfere with aerobic energy production, increasing the need for anaerobic glycolytic ATP production.

### Increased energy expenditure in persistent fatigue

Several findings point to behavioral-energy expenditure exceeding energy availability in patients with CFS and CRF. For example, one study reported that about half of a group of patients with CFS had perceived energy expenditure levels that exceeded their perceived energy availability (Jason et al., 2009). In addition, a non-pharmacological intervention aimed at decreasing fatigue in patients with CFS was effective only in those patients in whom perceived energy availability and expenditure were matched (Brown et al., 2011). In CRF, we have recently shown that cancer survivors reporting more-severe fatigue exhibited an increased tendency to exert effort (Lacourt et al., under review). Cancer patients actively undergoing treatment did not show this association, but rather showed a decreased inclination for exerting effort. Similarly, Mortimer et al. (2017) reported significant *positive* associations between fatigue severity and average daily caloric expenditure in breast cancer patients after the fourth cycle of chemotherapy. Thus, there is preliminary evidence suggesting that energy expenditure does not match (perceived) energy availability.

### Alterations in circadian rhythms and sleep in persistent fatigue

As mentioned above, low-grade inflammation-induced changes in metabolism can be mediated by changes in circadian rhythms and sleep, possibly through changes in melatonin rhythmicity (Box 2). Below, we discuss the findings on sleep, circadian rhythm, and melatonin in relation to chronic fatigue (see also Table 3).

Box 2 **Melatonin.**

Melatonin moderates both sleep and circadian rhythm and an important biomarker for sleep and circadian rhythm. Melatonin (*N*-acetyl-5-methoxytryptamine) is a hormone which moderates both sleep and circadian rhythms and is specifically known for its importance in sleep onset. Melatonin concentrations show a circadian pattern with slowly increasing levels during the day, leading to maximal levels at the time of sleep onset, followed by a slow decrease until it reaches a minimum level in the morning when it is time to wake up. In addition to its function for sleep, melatonin can also act as an antioxidant and immune regulator, playing a role in mitochondrial DNA protection (Ramis et al., 2015). Studies in rodents confirm that inflammation and stress are capable of modulating melatonin levels (Persengiev et al., 1991; Huang et al., 2014) and that melatonin can reduce inflammation and oxidative stress associated with sleep deprivation (Kim et al., 2012; Zhang et al., 2013). However, melatonin administration may be insufficient to restore sleep-wake rhythmicity (Mirmiran and Pevet, 1986).

**Table 3:** Overview of discussed studies on the association between fatigue and sleep/circadian rhythm.

**References**	**Study design and sample description**	**Description**	**Relevant results**
**OBSERVATIONAL STUDIES**			
**CFS**			
Russell et al., 2016	Prospective diary and actigraphy study; Patients with CFS (*n* = 27) followed for 6 days.	Diaries captured subjective sleep and presleep arousal, mood, and fatigue; Actigrahy data was used to capture sleep efficiency and sleep fragmentation.	Subjective sleep predicted following-day fatigue. Actigraphy-captured sleep quality measures did not predict following-day fatigue. *As no control group was included, it is unclear whether these associations are specific for patients with CFS*.
Guilleminault et al., 2006	Cross-sectional observational study; Patients with CFS but without reported sleepiness (*n* = 14) vs. healthy controls (*n* = 14).	Patient-reported sleep and sleep disruptions; EEG output of one night to capture duration and frequency of sleep cycles and respiratory measures.	Patients with CFS more often reported disrupted sleep. EEG output indicated several subtle differences in the CFS group compared to the controls indicative of abnormal sleep progression and NREM sleep instability. *As patients with CFS also showed increased respiratory effort and nasal flow limitation, the authors argue that abnormalities in sleep progression might be due to underlying undiagnosed apnea*.
Milrad et al., 2017	Cross-sectional study; Patients with CFS (*n* = 60)	Patient-reported sleep and fatigue in association with plasma levels of inflammatory mediators IL-6, TNF-a, and IL-1B.	Greater fatigue severity was associated with worse sleep quality and increased inflammation. Reduced sleep quality was in addition related to increased inflammation. *Associations between fatigue and inflammation were not reported. As no control group was included, it is unclear whether the reported associations were specific for CFS*.
Hamilos et al., 2001	Cross-sectional study; patient with CFS (*n* = 10) vs. controls (*n* = 10)	Circadian rhythm of body temperature assessed in 5-min intervals over 48 h.	Circadian rhythms did not differ between CFS and controls. There was a tendency for greater variability on rhythm in the CFS group. *The authors conclude that disturbance in body temperature circadian rhythm is an unlikely cause of CFS symptoms*.
Rahman et al., 2011	Cross-sectional study; Patients with CFS (*n* = 15) vs. controls (*n* = 15)	Group comparisons on diurnal cortisol concentrations (assessed for one day), circadian rhythm, sleep efficiency and fragmentation (from actigraphy data assessed over 5-days), and self-reported activity and symptoms in 5-day diary assessment.	Patients and controls did not differ in diurnal cortisol patterns or concentrations, circadian rhythm, or objective sleep measures. Patients with CFS reported poorer sleep quality.
Williams et al., 1996	Cross-sectional study; Patients with CFS (*n* = 20) vs. controls (*n* = 17)	Group comparisons of circadian rhythm (24-hr continuous body temperature; patient-reported physical activity levels in 30 min intervals), and dim light melatonin onset (DLMO; time of first rise in melatonin between 18:00 and 24:00).	Groups did not differ in circadian rhythm (of body temperature) and timing of DLMO. DLMO and peak in body temperature (acrophase) were associated in controls, but not in CFS patients.
Knook et al., 2000	Cross-sectional study; Adolescents with CFS (*n* = 13) vs. controls (*n* = 15)	Group comparisons on self-reported sleep onset and duration, sleep quality and sleep problems as well as changes in salivary melatonin concentrations between 17:00 and 02:00.	Adolescents with CFS more often reported unrefreshing sleep, nocturnal wake-ups, and restless sleep. Salivary melatonin level increased during testing in both groups; the increase was stronger in the CFS group resulting in higher levels between midnight and 02:00 a.m.
**CRF**			
Miaskowski et al., 2011	Cross-sectional study; Cancer patients planned for radiation therapy (*n* = 185; mixed group of breast, prostate, lung, or brain cancer)	Correlations between self-reported sleep disturbances and fatigue as well as objective assessment of sleep quality and circadian rhythm through actigraphy prior to onset of radiation therapy.	Small to moderate associations were found between higher patient-reported fatigue and patient-reported poorer sleep. Some small correlations were found between fatigue and objective measures of sleep and between fatigue and circadian rhythm (acrophase: the clock time of the peak amplitude was later in patients with higher fatigue).
Liu et al., 2012a,b	Longitudinal observational study; Breast cancer patients scheduled to receive (neo)adjuvant chemotherapy (*n* = 97); data on inflammation was available in a subset (*n* = 53).	Correlations between changes in fatigue, objective/subjective sleep, and inflammation during chemotherapy with assessments made at baseline and during chemotherapy cycle 1 and 4. Objective sleep parameters were obtained from actigraphy data. Associations between fatigue and sleep with inflammation were reported in a second paper (Liu et al., 2012b).	Fatigue increased during chemotherapy and was associated with reported sleep disturbances and some objective markers of sleep. Within-time point associations were moderate for fatigue with subjective sleep measures and mostly absent for objective sleep measures (Liu et al., 2012a) Inflammation increased over time and was associated with increases in both fatigue and poorer reported sleep (Liu et al., 2012b).
**CLINICAL TRIALS**			
**CFS**			
Williams et al., 2002	Within-subjects randomized controlled clinical trial; Patients with CFS (*n* = 42)	Effects of melatonin (5 mg/night for 12 weeks) and phototherapy (30 min of bright light therapy/morning for 12 weeks) on body temperature circadian rhythm, melatonin secretion profiles and several patient-reported outcomes. The order of melatonin and phototherapy intervention was randomized and each intervention was preceded by 12 week of placebo.	Neither intervention affected patient-reported symptoms, including fatigue and sleep disturbances. The interventions also did not affect circadian rhythm (with the exception of a slight change in acrophase in the phototherapy intervention) or DLMO. *Of the 42 participants who were randomized, only 30 completed the study*.
van Heukelom et al., 2006	Clinical trial; 29 patients with CFS and late DLMO (>21:30)	Effects of melatonin (5 mg per day, 5 h before DLMO for 3 months) on patient-reported fatigue. Fatigue was assessed before and after treatment.	Melatonin treatment decreased fatigue. This effect was driven by patients with later DLMO (>22:00, *n* = 21), as scores did not improve in those with relatively early DLMO (*n* = 8). *Study lacks placebo-control condition*.
**CRF**			
Ancoli-Israel et al., 2012; Neikrug et al., 2012	Randomized controlled trial; Breast cancer patients undergoing chemotherapy (*n* = 39) randomized in 2:1 ratio	Effects of 30 min of morning exposure to bright white light (BWL) vs. dim red light (DRL) (placebo) therapy throughout the first 4 cycles of chemotherapy on circadian rhythms (captured with actigraphy over 3 consecutive days) and patient-reported fatigue. Assessments were made at baseline, during treatment in cycle 1 and 4, and during recovery after cycle 1 and 4.	BWL protected against the reductions in activity and rhythmicity as well as increases in fatigue that were observed in the DRL group; changes in fatigue were not mediated by or associated with changes in sleep or circadian rhythms. *Fatigue was increased in the DRL group only during treatment and not in the recovery assessments, while circadian rhythmicity changes were more persistent, suggesting that the observed changes in fatigue were very transient and not reflective of more persistent fatigue that is reported by some breast cancer patients*.
Redd et al., 2014	Randomized controlled trial; Cancer survivors (*n* = 36; mixed diagnoses) up to 3 years post primary cancer treatment randomized in 1:1 ratio	Effects of 30 min of morning exposure to BWL or DRL for 4 weeks on patient-reported fatigue.	BWL lead to consistent improvements in fatigue with lowest fatigue at last assessment, 3 weeks after completion of the intervention end point. DRL led to an improvement in fatigue in week 2 of the intervention, followed by an increase back to baseline. *Effects could not be explained by changes in depression*.
Johnson et al., 2017	Randomized controlled trial; Cancer survivors (*n* = 81; mixed diagnoses) having completed primary cancer treatment randomized in 1:1 ratio	Effects of 30 min of morning exposure to BWL or DRL for 4 weeks on patient-reported fatigue.	BWL led to consistent improvements in fatigue whereas DRL led to some improvement only up to week 2.

In CFS, poor sleep and altered melatonin rhythmicity, but not alterations in circadian rhythmicity have been reported. Patients with CFS report more sleep disturbances (Russell et al., 2016) and display abnormal sleep progression (i.e., greater cyclic alternating pattern rate) (Guilleminault et al., 2006). In addition, fatigue severity was associated with patient-reported poor sleep (Milrad et al., 2017), which was in turn associated with minor increases in plasma levels of the proinflammatory cytokines interleukin-1β and tumor necrosis factor-α. While no evidence has been found for altered circadian rhythm in CFS (Hamilos et al., 2001; Rahman et al., 2011), there is evidence for altered melatonin rhythmicity. For example, significantly higher nocturnal salivary melatonin levels were seen in adolescents with CFS who report unrefreshing sleep compared to healthy subjects (Knook et al., 2000). Further, the association between body temperature circadian rhythm and melatonin onset observed in healthy individuals was absent in patients with CFS (Williams et al., 1996). Although treatment with melatonin or phototherapy (daylight therapy) did not alleviate fatigue in CFS patients in one study (Williams et al., 2002), melatonin treatment was successful in reducing fatigue when participants were selected for a later-than-usual evening-melatonin onset (van Heukelom et al., 2006). The latter study did not include a placebo-control but fatigue reductions were significantly more pronounced in patients with very late onset vs. relatively early onset, suggesting that the effects are not likely explained by placebo effect alone.

In cancer patients, associations between CRF and disrupted circadian rhythm and sleep have been reported before, during, and after cancer treatment (Miaskowski et al., 2011; Payne, 2011; Ancoli-Israel et al., 2014). In a longitudinal study of breast cancer patients, increases in fatigue during chemotherapy were related to increased reports of sleep disturbances as well as increased nap time and decreased wake time during the day (Liu et al., 2012b). In the subset of these patients, for whom information on inflammation was available, increases in either fatigue or disturbed sleep were associated with increased inflammation (Liu et al., 2012a). Interventions aimed at normalizing circadian rhythm have proven successful in alleviating CRF. Bright white light therapy was effective in both preventing and treating circadian-rhythm desynchronization in patients with breast cancer (Neikrug et al., 2012) and improved fatigue in breast cancer survivors (Redd et al., 2014) and in a mixed sample of nonmetastatic cancer survivors (Johnson et al., 2017). Light treatment during chemotherapy for breast cancer was shown to prevent increases in fatigue, although surprisingly, these effects were not mediated by concurrent effects on circadian rhythm (Ancoli-Israel et al., 2012).

In summary, disturbed sleep and altered circadian or melatonin rhythmicity occur frequently among individuals with persistent fatigue, although persistent fatigue can be experienced in absence of sleep and circadian-rhythm alterations. Only a few studies have assessed the associations between low-grade inflammation and sleep or circadian rhythm in the context of fatigue, and the results are inconsistent. In addition, associations between sleep or circadian rhythm and metabolic outcomes have not been studied in fatigued populations.

## Summary and conclusion

We have here proposed a model of persistent fatigue as a consequence of chronic low-grade inflammation leading to an imbalance in energy availability and expenditure, which can be mediated and maintained by changes in circadian rhythms and sleep. The here discussed literature indeed points to associations between persistent fatigue in CFS and CRF with alterations in cellular metabolism, disturbed sleep, and, to a lesser extent, disruptions in circadian rhythm. For both CRF and CFS, multi-causal models are generally suggested (Papadopoulos and Clear, 2011; Moss-Morris et al., 2013; Bower, 2014); not only including low-grade inflammation and disturbed sleep, but also alterations in stress physiological responses, genetic vulnerability, and sociodemographic factors, among others. These causes are not mutually exclusive and feasibly point to a more limited number of final end points. We have here described such a final common pathway, incorporating several recognized contributors to persistent fatigue.

There are several limitations to the current state of knowledge. First, most studies have been carried out in patients with CFS patients, and this is especially the case for studies on metabolism and energy production. It is thus far unclear to what extent findings on metabolism and energy production also pertain to patients with CRF. Further, few reports are available that included more than one of the suggested parameters of our proposed model. Only two studies explored associations between low-grade inflammation and sleep, one in CFS patients and one in fatigued breast cancer patients. To the best of our knowledge, no studies have been published that describe associations between inflammation and metabolism in patients with persistent fatigue. Despite this lack of more-encompassing studies, the evidence thus far suggests that *reduced cellular-energy availability* might play a role in chronic fatigue when the fatigue is associated with *low-grade inflammation*. In addition, there is limited evidence *for increased behavioral-energy expenditure* suggesting that energy expenditure is not adapted to the level of fatigue or of energy availability.

Intervention studies aimed at improving aspects of the model presented, with the ultimate goal to alleviate fatigue, are still scarce. Nevertheless, it is already apparent that participants for such studies should be selected carefully and not solely on the basis of their fatigue experience. This is nicely illustrated by the study by van Heukelom et al. (2006), in which careful selection of individuals with CFS *with* later-than-usual evening-melatonin onset revealed significant beneficial effects of melatonin supplementation. Such effects were not observed when participants were selected solely on the basis of their fatigued state (Williams et al., 2002).

In sum, more research is needed to piece together the puzzle explaining how chronic low-grade inflammation can lead to the experience of persistent fatigue. Nevertheless, reduced cellular energy paired with increased or maladaptive changes in energy expenditure poses a potentially important explanation. Evidence for this model can be found for both low-grade inflammation and fatigue.

## Author contributions

TL, EV, and CH: contributed to conception of the model proposed in the review; TL: wrote the first draft of the manuscript; EV and GC: wrote sections of the manuscript; RD and CH: reviewed the manuscript at several stages and contributed to fine-tuning of the ideas presented in the review. All authors contributed to manuscript revision, read, and approved the submitted version.

### Conflict of interest statement

The authors declare that the research was conducted in the absence of any commercial or financial relationships that could be construed as a potential conflict of interest.
